# Revisiting the Roles of Pro-Metastatic EpCAM in Cancer

**DOI:** 10.3390/biom10020255

**Published:** 2020-02-07

**Authors:** M. Aiman Mohtar, Saiful Effendi Syafruddin, Siti Nurmi Nasir, Low Teck Yew

**Affiliations:** UKM Medical Molecular Biology Institute (UMBI), The National University of Malaysia, Cheras 56000, Kuala Lumpur, Malaysia; effendisy@ppukm.ukm.edu.my (S.E.S.); ctnurminasir@ppukm.ukm.edu.my (S.N.N.); lowteckyew@ppukm.ukm.edu.my (L.T.Y.)

**Keywords:** cancer, circulating tumour cell, adhesion, metastasis, biomarker

## Abstract

Epithelial cell adhesion molecule (EpCAM) is a cell surface protein that was discovered as a tumour marker of epithelial origins nearly four decades ago. EpCAM is expressed at basal levels in the basolateral membrane of normal epithelial cells. However, EpCAM expression is upregulated in solid epithelial cancers and stem cells. EpCAM can also be found in disseminated tumour cells and circulating tumour cells. Various OMICs studies have demonstrated that EpCAM plays roles in several key biological processes such as cell adhesion, migration, proliferation and differentiation. Additionally, EpCAM can be detected in the bodily fluid of cancer patients suggesting that EpCAM is a pathophysiologically relevant anti-tumour target as well as being utilized as a diagnostic/prognostic agent for a variety of cancers. This review will focus on the structure-features of EpCAM protein and discuss recent evidence on the pathological and physiological roles of EpCAM in modulating cell adhesion and signalling pathways in cancers as well as deliberating the clinical implication of EpCAM as a therapeutic target.

## 1. Introduction

Epithelial cell adhesion molecule (EpCAM) is a type-1 transmembrane glycoprotein that was first discovered 40 years ago from monoclonal antibody screening against antigens derived from colorectal cancer cells [1]. Since then, many studies have further corroborated that EpCAM is indeed an immunogenic molecule that can be targeted by monoclonal antibodies especially in cancers. As a result of its multiple discoveries as an antigen-responsive moiety, EpCAM has received several aliases such as 17-1A, 323/A3, 311-1K1, Ber-EP4, gp 38, CD326, EGP-2, EGP34, EGP40, ESA, Ep-CAM, GA733-2, HEA125, KS1/4, KSA, AUA1, MH99, MK-1, FU-MK-1, MOC31, TACSTD-1, G8.8* and TROP1 [2,3]. However, it has reached to a consensus that EpCAM should be adopted as the standard nomenclature [4].

EpCAM has been studied in a wide-variety of human carcinomas and normal epithelial tissues and it is considered to be the most studied tumour-associated antigen [4]. The expression of EpCAM is significantly elevated in a number of human adenocarcinomas as well as squamous cell carcinomas [5]. Due to the overexpression of EpCAM in cancer and its accessibility on the cell surface, EpCAM is of high interest to be developed as a marker for prognosis, diagnosis and therapeutic intervention for epithelial cancers. Numerous therapeutic strategies have been developed to target EpCAM and several of them are currently undergoing clinical trials [4,6,7].

In this review, we focus on the structural features of the EpCAM gene and protein as well as its post-translational modifications and relate them in the context of cancer. We will also discuss the current updates on the roles of EpCAM especially in cell adhesion, cell signalling and additional insights on EpCAM functions in the cancer-related landscape.

## 2. EpCAM Structure and its Oligomeric State

### 2.1. Gene and Protein Architecture

**Figure 1:**
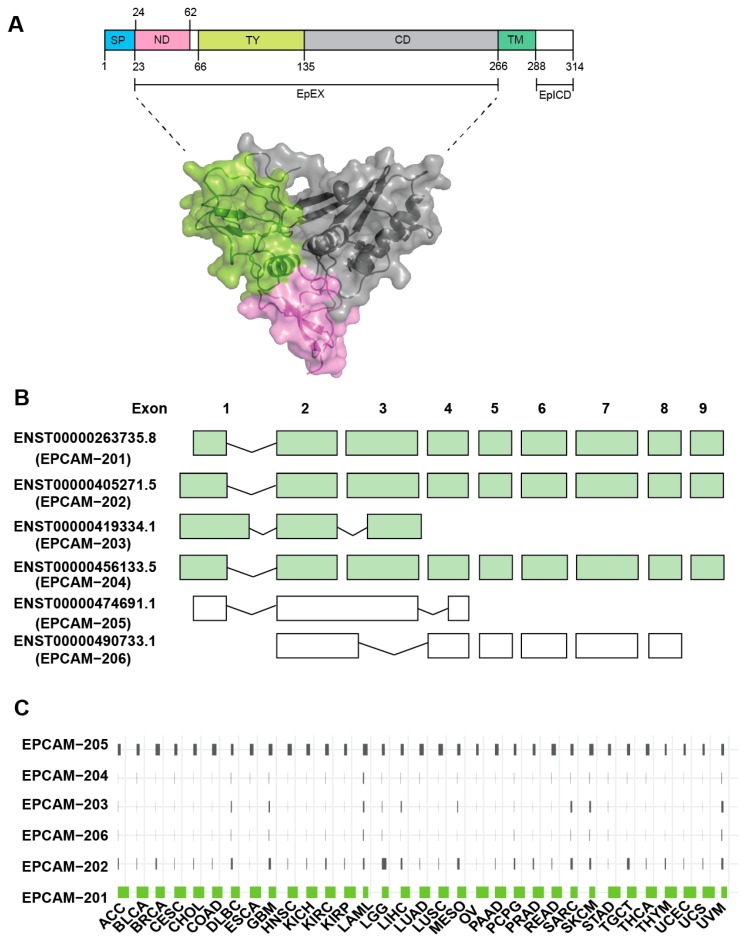
Epithelial cell adhesion molecule (EpCAM) protein structure and splice variant expression in cancer. (**A**) The secondary structure of EpCAM which consists of signal peptide (SP, blue), N-domain (ND, pink), Thyroglobulin type-1 domain (TY, lime green), C-domain (CD, grey), transmembrane domain (TM, grey) and intracellular part (EpIC, white). Three-dimensional illustration and surface representation of the EpCAM cleaved extra-cellular domain (EpEX) (PDB code: 4MZV) color-coded as in the secondary structure. (**B**) Schematic of EpCAM gene structure and the splice variants extracted from Ensembl database (http://www.ensembl.org). The predominant isoform, EpCAM-201, consists of 9 exons. Isoforms color-coded in green are those encode for EpCAM protein. (**C**) Bar-plot shows the commonly expressed EPCAM isoforms (from 0% to 100%) across the TCGA-Pan-cancer analysis.

The human epcam gene (*hepcam*) comprises of 9 exons and is located on chromosome 2p21 that spans ~42kb in genomic region. This gene is translated into EpCAM protein that consists of 314 amino acids, which constitute ~40kDa in molecular weight (Figure 1A). The epcam gene can give rise to 6 transcript variants which include *EpCAM-201*, *EpCAM-202*, *EpCAM-203*, *EpCAM-204, EpCAM-205* and *EpCAM-206* (Figure 1B). *EpCAM-201* is the predominant isoform which is corroborated by the TCGA large scale cancer transcriptomic findings (Figure 1C). This isoform is hereinafter referred to as EpCAM. Interestingly, the expression of *EpCAM-205* is also notable across all cancer types (Figure 1C) even though this particular isoform is annotated not to get translated into functional protein (Figure 1B). This *EpCAM-205* may get transcribed and processed in cancer but subsequently undergo post-transcriptional degradation. Moreover, perhaps this *EpCAM-205* spliced variant could play direct roles in regulating tumourigenesis as observed in other genes [8,9]. However, this is only a working hypothesis and further investigations on the function of *EpCAM-205* are required to support this claim.

DNA hypomethylation at the EpCAM promoter region has been frequently observed in several cancer types such as in colorectal [10], ovarian [11,12] and breast cancer [13]. There was an inverse correlation between EpCAM expression level and the EpCAM promoter DNA methylation status in these cancer types. Moreover, in the ovarian cancer EpCAM negative cells, repressive histone marker such as H3K27me3 was also found at the EpCAM gene regulatory elements [12]. These observations demonstrate that the regulation of EpCAM expression in cancers seems to be controlled at the epigenetic level. Several transcription factors were identified to bind the EpCAM gene regulatory elements that include the ETS family and SP1 transcription factors [14]. Moreover, study in hepatocellular carcinoma reported that EpCAM expression in this cancer type is regulated by the WNT signalling pathway via its downstream transcriptional effectors, TCF and Lef1 [15].

Structurally, the full-length EpCAM protein can be divided into four essential parts (Figure 1A). The first part consists of a stretch of signal peptide (Met1-Ala23) located at the N-terminal of EpCAM that is cleaved off during synthesis. Therefore, the amino acid sequence for a mature EpCAM protein starts only at Gln24. An alternative shorter signal peptide can exist which can be cleaved off by signal peptidase at Ala21 [16]. The second part of EpCAM stretches from Gln24-Lys265. This region forms the EpCAM ectodomain, which is also called EpCAM cleaved extra-cellular domain (EpEX) [17]. Following the EpEX region is the single-pass transmembrane region that encompasses Ala266 to Ile288. Finally, extending from Ser289 to Ala314 is a short cytoplasmic domain, consisting of only 26 aa. This cytosolic region is termed EpCAM cleaved IntraCellular Domain (EpICD).

The EpEX domain is rich in cysteine residues (12 cysteines) [2]. There are several conformation models of EpCAM in regard to disulphide arrangement [16,18,19]. The latest model suggested an assignment of intramolecular disulphide linkages that resembles the thyroglobulin (TY) type 1A domain [2,16]. The EpEX domain can undergo proteolytic cleavage, for example at Arg80 and Arg81 under non-reducing condition, but the resulting N-terminal cleavage peptide can still be linked together on the same parent molecule via disulphide linkage (Cys66-Cys99) [20,21,22]. Furthermore, the cysteines in this region suggest multiple disulphide linkages can be formed with client proteins during its protein trafficking or for its extracellular oncogenic function. There are additional cleavage events that will be discussed below.

### 2.2. EpCAM Functional Domains and Motifs

The name EpCAM was coined based on its epithelial of origin and was found to promote cell adhesion by interacting with other EpCAM molecules on the neighbouring cells (homophilic interaction) [23]. In general, proteins that promote cell-cell adhesion belong to transmembrane cell adhesion molecules (CAMs) which can be further divided into four families: cadherins, integrins, selectins and the immunoglobulin superfamily (IgSF) [24,25,26]. These group of proteins are responsible for maintaining the integrity of tissue architecture. However, EpCAM does not belong to any of this family as EpCAM is structurally different from these CAM proteins [3]. The homophilic interaction mediated by EpCAM is relatively weak compared to those mediated by the CAM proteins. A study using confocal and electron microscopy have revealed that EpCAM did not form any type of adherent junctions and partially colocalize with E-cadherins, a major type of adhesion molecule of epithelial cells, during cell-cell contacts [18]. However, ectopic expression of EpCAM was able to bring two neighbouring cells into close proximity. This suggests that EpCAM partakes a different mechanism of cell adhesion.

The crystal structure of EpEX was recently deciphered, confirming some of the predicted structural domains of EpCAM [27]. It is important to note that the protein used in the study has been mutated to prevent N-linked glycosylation of Asn74, Asn111 and Asn198 residues. The N-terminal EpEX domain can be subdivided into three functional domains: i) The N-terminal cysteine-rich domain (ND), ii) the thyroglobulin type 1A domain (TY Domain) and iii) the carboxy-terminal domain, C-domain or CD. The ND domain is most similar to the WW protein module instead of the previously thought epidermal growth factor (EGF)-like motif (aa 27-59) [2]. Previously, an EGF-like motif was assigned to aa 66-135 of EpCAM. However, it was discovered later that the disulphide-bonding patterns of this region resemble that of the thyroglobulin (TY) type 1A [16] rather than EGF-like domain; and this was confirmed with EpEX crystal structure [27]. This TY region was thus assigned as a separate domain that forms a structural loop that is stabilized by three disulphide linkages. Lastly, the TY domain is followed by the CD domain that is cysteine-free and the crystal structure demonstrates that it belongs to the α+β fold class. Although this structure is not well-characterized, the fact that it is cysteine-poor, it may provide some flexibility in the extracellular environment due to its low electron density. The next structural element is the 23 aa single-pass transmembrane domain that interacts with Claudin-7 where this complex is suggested to promote tumourigenesis [28]. This is followed by a 26aa cytoplasmic domain EpICD [17]. This domain is responsible for anchoring itself to the cytoskeleton alpha-actinin during cell-cell contacts [29]. It also contains a PDZ binding site but the interaction with the multi-PDZ domain has not been studied yet [2].

### 2.3. EpCAM Oligomeric Structure

An important feature of EpCAM is that it can exist in multimers, suggesting that it may possess different functions at different physiological conditions. The oligomeric structure is a feature of adhesion complex formed between two EpCAM molecules on different cells [27,29,30]. It was found that EpCAM can form cis-dimer and this was a predominant form of EpCAM in solution [27]. EpCAM can also form a weak tetramer, especially at the cell surface, most likely formed by intercellular interaction between cis-dimers on opposing cells, mediating cell-cell adhesion [30]. Another study has suggested that an alternative tetramer formation which can be pre-formed at cell surface on the same cell (lateral interaction) [29]. Hence, this cis-tetramer can form the trans-octameric structure on EpCAM in the neighbouring cells. The establishment of EpEX crystal structure supports the first model suggesting that EpCAM has cis-dimer orientation, forming trans-tetramer with neighbouring cells [27]. Recent studies however, exerted new evidence in that EpCAM forms neither trans-tetramer nor trans-octamer with another EpCAM molecule on neighbouring cells which is capable of mediating cell-cell adhesion activity [31]. The cis-dimers are the prevalent form and surprisingly, high concentrations of EpCAM dimers tend to resist each other [27,31]. However, this is not overtly true due to limitations of the techniques used in the study that includes molecular dynamics modelling and chemical cross-linking coupled with mass spectrometry [31]. These approaches may not detect the presence of cross-linked higher-order oligomers and therefore it is possible that the trans-tetramer structure for example, exist but could not be identified.

## 3. EpCAM Post-Translational Modifications

### 3.1. Disulphide Bond

**Figure 2:**
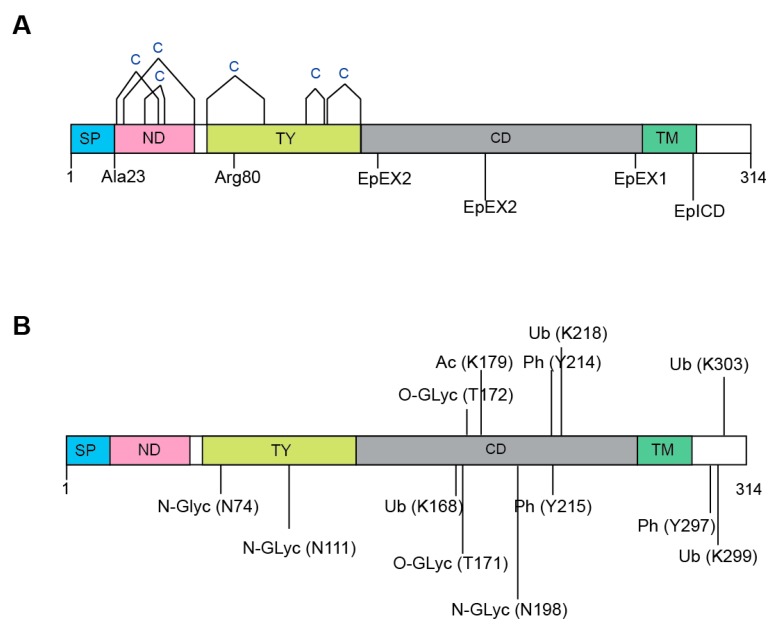
Post-translational modifications of EpCAM. (**A**) EpCAM harbours six disulphide bridges that cluster around the N-domain (ND) and Thyroglobulin type-1 domain (TY). Proteolytic cleavages can take place at two locations. i.e., after Ala23 or Ala21 by a signal peptidase; and in the TY-repeat, between Arg80 and Arg81. Additionally, EpCAM undergoes a two-step regulated intramembrane proteolysis (RIP), whereby EpCAM is first cleaved close to the plasma membrane at the EpEX1 site, leading to the shedding of the EpEX domain; followed by a second cleavage at the EpICD site. In another proposed RIP pathway, the first cuts are made to the ectodomain via consecutive cleavages at two sites (EpEX2), followed by the EpICD cleavage. (**B**) The presence of three N-glycosylation sites located on Asn74, Asn111 and Asn198 of EpCAM have been confirmed by numerous reports. Other post-translational modifications that have been discovered include: O-linked glycosylations at Thr171 and Thr172; phosphorylations at Tyr214, Tyr215 and Tyr297; ubiquitylations at Lys168, Lys 218, Lys 299 and Lys303; and acetylation at Lys179.

EpCAM undergoes extensive post-translational modifications. Altogether, EpCAM harbours twelve cysteine residues that are clustered around the N-Domain (ND) and Thyroglobulin type-1 domain (TY) (Figure 2A). These twelve cysteines form intra-molecular disulphide bridges along the N-Domain, i.e., Cys27-Cys45, Cys29-Cys59 and Cys38-Cys48; as well as the TY domain, i.e., Cys66-Cys99, Cys110-Cys116 and Cys118-Cys135 [2,16].

### 3.2. Proteolytic Cleavage

The functions and activity of EpCAM are highly dependent on proteolytic processing [2]. As aforementioned, the N-terminal signal peptide of EpCAM is first cleaved off after either Ala23 or Ala21 (in 1% of cases) by a signal peptidase for the purpose of the endoplasmic reticulum (ER) targeting during protein maturation [16,21,22] (Figure 2B). On the other hand, serine and cysteine proteases cleave EpCAM between Arg80 and Arg81 in the TY-repeat [16,19,32]. Among the cysteine proteases, the cathepsins are known to promote the metastasis of tumour cells by enhancing extracellular matrix degradation [33,34]; and EpCAM has been demonstrated to function as a membrane-bound protease inhibitor that can inhibit cathepsins either by binding to active site cleft of cathepsin via its TY domain; or serving as a “decoy” substrate whereby cathepsins can cleave the Arg80/Arg81 bond [35]. Since cathepsins are often expressed by metastatic cancer cells, overexpression of EpCAM can thus lead to stronger inhibiting effects that may protect these tumour cells against cathepsins during tumour progression [4].

### 3.3. Regulated Intramembrane Proteolysis (RIP)

Regulated intramembrane proteolysis (RIP) is a conserved signal-transducing mechanism that allows information to be transmitted across cellular compartments via a two-step cleavage process [36]. With RIP, EpCAM is first cleaved by metalloprotease tumour necrosis factor-alpha converting enzyme (TACE/ADAM17) close to the extracellular side of the plasma membrane, leading to the shedding of the extracellular domain of EpCAM named EpEX [17]. In the second step, presenilin 2 (PS-2), a protease component of the γ-secretase complex cleaves the C-terminal intracellular domain (EpICD), producing a 5 kDa peptide [17]. In 2013, Schnell et al. demonstrated an additional RIP pathway for EpCAM whereby consecutive cleavages at two sites within the cysteine-poor motif in EpCAM’s ectodomain, followed by intramembrane proteolysis, release the EpICD [35]. Following RIP, the EpICD fragment is translocated from the cytoplasm into the nucleus and form complex with FHL2, beta-catenin, and Lef-1, stimulating the expression of c-Myc and cyclin A/E genes [37]. This has been shown to drive cell proliferation, support hyperplastic growth and is oncogenic in immunodeficient mice [17,38,39].

### 3.4. N-linked Glycosylation

Altogether, an EpCAM protein harbours three extracellular N-glycosylation sites that are located on Asn74, Asn111 and Asn198 (Figure 2B) [19,20,32]. It has been demonstrated by Munz et al. that glycosylation at Asn198 is not only essential for maintaining the stability for EpCAM, but it also affects the expression level, as well as half-life of the molecule at the plasma membrane [20]. Additionally, EpCAM was found to be hyperglycosylated in carcinoma tissue as compared to autologous normal epithelial [31,40]. This observation was corroborated by another study that compared the levels of EpCAM glycosylation using biopsies obtained from 60 patients suffering from head and neck carcinomas, and 26 pairs of autologous healthy thyroid biopsies [40]. In this study, tumour-derived EpCAM was heavily glycosylated while EpCAM derived from autologous thyroid was not or weakly N-glycosylated.

In a recent study, all three EpCAM’s N-glycosylated Asn residues were mutated to Gln, and ectopically expressed in MCF-7 and MDA-MB-231 breast cancer cells [41]. It was shown that these mutations did not affect EpCAM in terms of expression level or membrane localization. Nevertheless, these mutants had significantly reduced the ability to promote epithelial to mesenchymal transition in breast cancer. Using the same deglycosylated mutant breast cancer cell lines, Zhang et al. showed that deglycosylation of EpCAM promoted apoptosis and inhibited cell proliferation [42]. Addition of 5-fluorouracil to the deglycosylated mutant cell lines enhanced the cell cytotoxicity, promoting apoptosis by downregulating the expression of the anti-apoptotic marker Bcl-2 and upregulating the expression of the pro-apoptotic proteins Bax and Caspase 3 [42]. EpCAM expression was associated with cell adhesion, and N-glycosylation mutation of EpCAM decreased adhesion capacity in breast cancer cells [43]. Therefore, it is of high interest to study glycosylation of EpCAM or perhaps other types of post-translational modifications that contribute to the onset of cancer.

### 3.5. Other Post-Translational Modifications

Due to the recent emergence of OMICS technologies, more post-translationally modified residues have been found on EpCAM, nonetheless the exact functions of such modifications have yet to be deciphered (Figure 2B). For example, two O-linked glycosylations have been found, i.e., Thr171 and Thr172, from two out of twelve human cell lines derived from different organs using the “bottom-up” proteomics approach [44]. Although phosphorylation is one of the most-studied PTMs, it is relatively uncommon in EpCAM [45]. It is only recently that Tyr214, Tyr215 and Tyr297 were found to be phosphorylated using a motif-targeting quantitative phosphoproteomic strategy. Additionally, Lys168, Lys 218, Lys 299 and Lys303 in EpCAM were found to be ubiquitylated [46]; while Lys179 was acetylated [47]. However, extra caution needs to be exercised with respect to the false discovery rate (FDR) of these identifications, in addition to the fact that these additional PTMs are unlikely to have undergone sufficient validation and follow-up.

## 4. Pathological and physiological EpCAM functions

### 4.1. EpCAM Expression in Normal Tissues

EpCAM is expressed in most epithelial cells and during embryogenesis [48]. It is localized at cell-cell boundaries, specifically at the basolateral membrane of pseudo-stratified and transitional epithelia [49,50]. EpCAM expression was not detected in normal squamous stratified epithelia. Early studies on EpCAM function have suggested that this molecule mediates cell adhesion in a Ca^2+^-independent manner by interacting with other EpCAM molecules on neighbouring cells (homophilic interaction) [18,23]. Later, it was shown that this homophilic interaction was rather weak which is via oligomerisation of EpEX in cis- and trans-interactions in the intercellular space [29,30]. When the two molecules engage, the intracellular part EpICD is thought to be anchored to the actin cytoskeleton α-actinin [29]. Additionally, EpCAM participates in the formation of functional tight junctions and regulates claudin dynamics, for example by directly binding to claudin-7 that together with EpCAM is responsible for maintaining intestinal lining integrity [28,51,52,53]. Further reports suggest that it interacts with several important CAMs and regulates adhesive properties between cells and cell-matrix, including tight junctions, adherens junctions, desmosomes and hemidesmosomes [52,53,54,55]. For example, EpCAM inhibits protein kinase C (PKC) via its EpICD tail that leads to aggravated myosin contractility and impairs cadherin-mediated cell-cell adhesion [56].

However, there is a debate on whether EpCAM plays a role as a cell adhesion mediator as its name suggested. For example, overexpression of EpCAM has a negative effect on the strength of classical CAM’s E-Cadherin-mediated cell adhesion where it weakens the property of adherens junctions [54,57]. Furthermore, CRISPR-Cas9 mediated EpCAM knockout in FaDu hypopharynx carcinoma cell line showed no notable effect on cell-matrix or cell-cell adhesion property [58]. Inhibition of cleavage activity also does not affect EpCAM cell adhesion property suggesting that EpCAM cell surface expression (EpEX) is intact upon RIP signalling. Since oligomerization is a feature of adhesion complex, the resolved EpCAM crystal structure does not suggest EpCAM forms a higher-order arrangement, particularly more than dimerization [27]. As described previously, a recent study suggested that EpCAM does not homo-oligomerize with another EpCAM on the other cell [31]. Since this oligomerization is a signature of homophilic interaction capable of mediating cell-cell adhesion activity, it is questionable to conclude that EpCAM has a direct role in homophilic adhesion molecule [31,58]. Of note, the closest homologue is TROP2, which shares 67% amino acid sequence similarity, hence one of EpCAM aliases is TROP-1 [59,60]. Fornaro et al. showed that neither TROP1 nor TROP2 have any roles as homophilic adhesion molecules but appear as signal transducers [59]. This suggests that there might be a window or stage that requires different functions of EpCAM, for example as a mediator of cell adhesion in early developmental stages of vertebrate embryos but later in adult, EpCAM is dispensable. Recent studies analysing the single-cell RNA-seq datasets showed that there was spatiotemporal patterning of EpCAM in mice embryonic development, where EpCAM is responsible for endodermal differentiation (e.g., lungs, colon epithelium) but was negatively regulated in mesodermal differentiation (e.g., heart) [61].

### 4.2. EpCAM in Cancer

**Figure 3:**
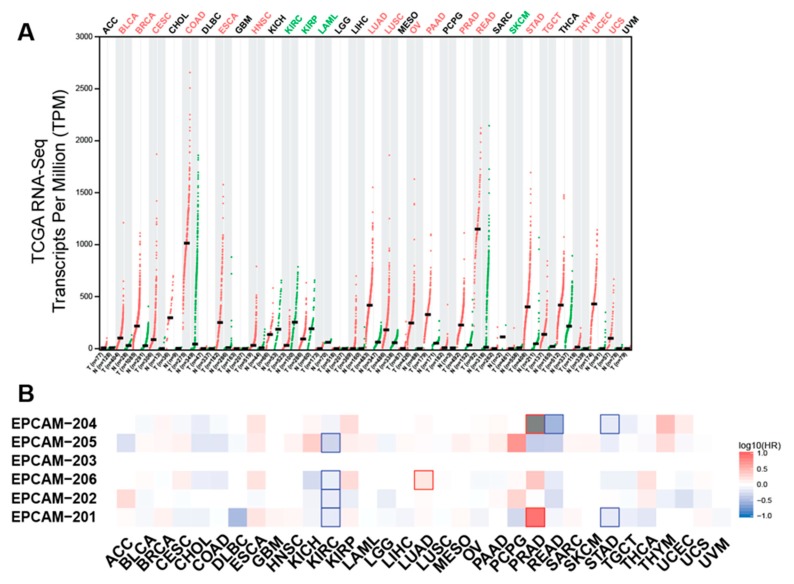
EpCAM mRNA expression in normal and cancer tissues and survival analysis. (**A**) Dot plots representing EpCAM expression, determined by RNA-Sequencing platform, in tumour and normal tissue samples from the TCGA and GTEx databases, respectively. The data were analysed and visualized using GEPIA2 web tool [65]. Cancer types that are labelled red have upregulated EpCAM expression compared to the normal tissues whereas cancer types labelled green are those that have lower EpCAM expression than their normal counterparts. (**B**) Heatmap showing the correlation between EpCAM gene isoforms expression and patients’ survival across cancer types from TCGA databases. The data were analysed and visualized using GEPIA2 web tool as in Figure 2A. The hazard ratios are in the logarithmic scale (log10); the red and blue blocks denote higher and lower hazard ratios, respectively. Blocks with highlighted border indicate statistically significant correlations.

As aforementioned, EpCAM is commonly observed to be upregulated in various primary tumour types and metastases, particularly on adenocarcinoma, certain squamous cell carcinoma and retinoblastoma [5,62]. Integration analysis of TCGA (cancer tissues) and GTEx (normal tissues) RNA-seq data showed that EpCAM is overexpressed in the majority of cancer types compared to paired normal tissues (Figure 3A). Increased EpCAM expression in patient tumour samples is associated with poor prognosis and therapeutic irresponsiveness [63,64]. The high expression of EpCAM in many cancer types would support cancer growth and progression because EpCAM involves modulating biological processes such as cell proliferation, differentiation, migration and invasion [2]. EpCAM roles in supporting tumourigenesis are discussed in the sections below.

### 4.3. EpCAM and Circulating Tumour Cells (CTCs)

Metastasis is the important step during cancer progression in which the metastasis process consists of several co-ordinated cascades; 1) dissociation of basal epithelial cells, 2) intravasation into circulation, 3) dissemination and evasion of immune cells inside the circulation, 4) extravasation at the distant organ and 5) micro-and macro metastasis growth [66]. The epithelial-to-mesenchymal transition (EMT) is a critical process during the metastatic cascade where the epithelial cancer cells undertake the mesenchymal properties and lose the epithelial phenotypes [67]. As a result, EMT process causes cells to have increased motility potential and invasiveness, thus enabling the cells to dissociate and extravasate into circulation as the circulating tumour cells (CTCs). The ability of these circulating tumour cells (CTCs) to survive inside the circulation and grow at the secondary site, in which the microenvironment might completely be different from the site of origin, are among the metastasis bottlenecks [66,68]. CTCs detection in blood has been widely used as a tool to diagnose many cancer types as well as prognostic markers to evaluate the disease’s aggressiveness and patients’ survival [69].

CTCs in the blood could be readily characterized by the presence of EpCAM, which as discussed previously, is the epithelial marker, and highly expressed in the cancers of epithelial origin [5]. Thus, the development of tools for CTCs detection is primarily based on exploiting the presence of EpCAM (along with other epithelial markers) on these CTCs [70]. CELLSEARCH®, however, is the only FDA-approved CTCs enumeration kit to date, which detects CD45-, EpCAM+ and cytokeratin 8, 18 and/or 19+ expressing CTCs in the blood [71]. Nevertheless, several studies have demonstrated either reduced or loss epithelial markers expression such as EpCAM and cytokeratin as the cancer cells undergo the epithelial-mesenchymal transition (EMT) during the metastasis process [72,73,74,75]. It has then been proposed that high expression of EpCAM observed in some CTCs might either due to the cells begin undergoing MET in the circulation prior to intravasation or simply due to cellular heterogeneities [76,77]. One major limitation with this EpCAM-dependent CTC detection approach is that it might not be sensitive to capture EpCAM^low^ CTCs and will completely disregard the EpCAM-CTCs, thus underestimating the CTCs number in the samples. For example, in breast cancer, CTCs can only be detected in about 60% of the metastatic breast cancer patients, indicating the presence of EpCAM-negative CTCs [78,79]. Thus, in order to overcome the CELLSEARCH® shortcoming, there have been EpCAM-independent CTCs capturing strategies developed [80,81]. The ability to detect and enumerate EpCAM low/negative CTCs are vital because these could facilitate for better predictions on the cancer stages, patients prognosis as well as designing the ideal therapeutic strategy and management of the patients care [79,82].

### 4.4. EpCAM and Cancer Stem Cells (CSCs)

A fraction of cancer cells may exhibit stem cell-like properties that are capable of undergoing self-renewal and differentiation [83]. This sub-population of cancer cells termed as cancer stem cells have been shown to possess enhanced tumour-initiating and metastatic capabilities, thus playing significant roles in driving tumourigenesis [83,84]. Several markers of cancer stem cells have been identified that include CD44, CD117 and CD133, which have been widely utilized for the characterization and downstream interrogation of these cancer stem cell population [85]. In addition, the tumour-initiating cells are also found to be enriched with EpCAM, thus classifying EpCAM as one of the cancer stem cell markers in several cancer types [86,87,88,89,90].

A recent study on breast cancer, for instance, has revealed that the EpCAM+ breast cancer cells have the propensity to self-renew and differentiate, and are more aggressive in-vivo as compared to their EpCAM-counterpart [91]. Furthermore, the EpCAM expressing ovarian cancer stem cells have increased colony and tumour formation capacities and are irresponsive towards doxorubicin and cisplatin treatments [92]. In line with these observations, the presence EpCAM+/CD44+ cancer stem cells in the colorectal cancer patient tissues is significantly correlated with more aggressive and higher tumour grade [93], further highlighting the critical role of EpCAM and the cancer stem cells in promoting tumour progression and survival as well as conferring resistant towards chemotherapeutic agents. Therefore, specific targeting or ablation of these EpCAM expressing cancer stem cells could be developed as novel cancer therapeutic strategies. In fact, there is an EpCAM/CD3-bispecific antibody developed, MT110, that functions to redirect T cells to target the stem cells [94,95]. This antibody has shown promising results in targeting colorectal and pancreatic cancer stem cells that led to impaired tumour growth in vivo [94,95].

### 4.5. EpCAM and Exosomes

Exosomes are secreted lipid bilayer extracellular vesicles that play role in cell communication by transporting macromolecules such as nucleic acids, proteins, and lipids between the cells [96]. Primary tumours and CTCs can also secrete exosome that carry pro-oncogenic molecules involve in promoting tumour growth, modifying the tumour microenvironments, establishing pre-metastatic niches and evading immune surveillance [97,98]. Interestingly, exosome secreted by the human colorectal cells-derived organoids also express EpCAM on their surface, thus enabling their isolation using anti-EpCAM-coupled magnetic beads [99]. In line with this observation, EpCAM is also found to be enriched in the exosomes isolated from the blood plasma and ascites fluid of ovarian cancer patients [100,101,102]. Little is known about the mechanism that regulates the EpCAM presence on the exosome surface. However, EpCAM is known to be highly expressed on the tumour types of epithelial origin [5]. Hence, one possible explanation is that during exosome biogenesis, the invagination of the plasma membrane might lead to the incorporation of EpCAM molecule on the formed endocytic vesicle [103].

One study has demonstrated that the level of EpCAM+ exosome is positively correlated with the ovarian cancer stage and aggressiveness [102]. In addition, blood plasma of colorectal cancer and lung cancer patients have a higher level of EpCAM+ exosome as compared to the blood plasma of their respective healthy controls [104,105]. Nonetheless, in contrast to ovarian cancer and colorectal cancer, EpCAM was absent in the exosome isolated from the breast cancer patients’ serum [101]. It has been suggested that this contrasting observation was due to the EpCAM being cleaved from the exosome surface. Nevertheless, since EpCAM is found to be enriched on the circulating exosomes in several cancer types, utilization of specific antibody against EpCAM has been among the strategies developed to detect and isolate exosomes for downstream investigation [102,106,107].

## 5. EpCAM Downstream Targets in Cancer

The WNT signalling pathway transcriptionally regulates *EpCAM* expression in hepatocellular carcinoma via its downstream effector TCF/Beta-catenin complex [15]. Much emphasis has been put to understand the roles of EpCAM regulated intermembrane proteolysis and cytoplasmic EpICD in promoting tumourigenesis. To date, the proposed mechanism on how EpICD could promote tumourigenesis seems to be well established [17]. In colorectal cancer for example, the proteolytic cleavage of membrane-bound EpCAM leads to the liberation of its intracellular domain (EpICD) into the cytoplasm [17]. This cytoplasmic EpICD subsequently form a transcription complex with FHL2, Beta-catenin and Lef-1, which are the downstream effectors of WNT signalling pathway, and activate the expression of pro-oncogenic genes such as *Myc* and the cell cycle regulators *cyclin* family [17]. Therefore, a high abundance of EpCAM in cancer will result in more EpICD being released into the cytoplasm, thus upregulating the expression of these important cell cycle regulators that will subsequently promote cell growth and tumourigenesis.

In addition to promoting cell proliferation, the roles of the WNT signalling pathway in regulating the expression of genes associated with cell stemness maintenance, self-renewal and differentiation have been well-established [108]. In the context of EpCAM, the shedding of EpICD from the membrane and translocation into the nucleus has been demonstrated to modulate the expression of cell stemness-associated genes via its binding to downstream effectors of the WNT signalling pathways [109]. This further highlights the important crosstalk between EpCAM and WNT signalling pathway in supporting tumourigenesis. This observation was also in agreement with EpCAM high expression in the cancer stem cells in which the EpICD function could be necessary to maintain the stemness properties of these cells. Overall, there is a positive feedback loop between EpCAM, via the cytoplasmic EpICD, and the WNT signalling pathway in promoting tumourigenesis.

**Figure 4:**
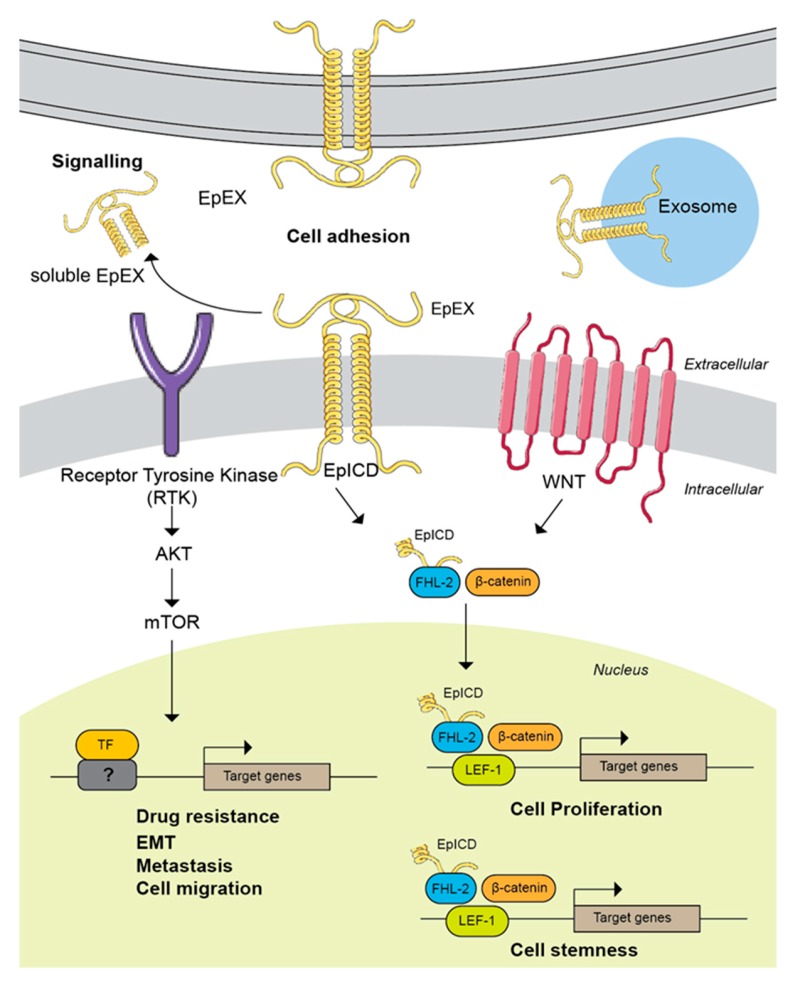
EpCAM localization and modulation of its downstream targets in cancer. The membrane bound EpCAM undergoes regulated intermembrane proteolysis that leads to the secretion of solubilised extracellular domain, EpEX and the liberation of cytoplasmic intracellular domain, EpICD. The cytoplasmic EpICD forms transcriptional complex with WNT signalling downstream effector to activate genes that are associated with cells proliferation and stemness maintenance. The solubilized EpEX acts as ligand to stimulate the oncogenic signalling pathways like the PI3K/AKT/mTOR via its binding to receptor tyrosine kinase and activate the expression of genes involve in supporting cancer cells growth and fitness. The membrane bound EpCAM can also function in promoting cell adhesion as well as being expressed on the exosome surface to mediate cell-cell communication.

Along with the cytoplasmic EpICD, the role of cleaved EpEX in supporting tumourigenesis has also started to gain attention. Upon EpCAM regulated intermembrane proteolysis, the solubilized extracellular domain could act as a ligand for receptor tyrosine kinases (RTKs) and activate the several oncogenic signalling pathways that would in turn support cancer cells growth and fitness [110]. A study by Liang et al. have shown that EpEX activates the ERK1/2 signalling pathway via its binding to EGFR, thus promoting colorectal cancer cell proliferation and migration [110]. The ability of EpEX to act as a ligand for EGFR is in line with the fact that EpCAM N-terminal domain (a region that constitutes the solubilized EpEX) contains the EGF-like motif, thus possibly enabling it to interact and bind to EGFR. Moreover, EpCAM has also been reported to promote tumourigenesis in prostate [63,111] and nasopharyngeal cancer [112] by modulating the activity of PI3K/AKT/mTOR pathway. These studies, however, did not further investigate whether the modulation of the PI3K/AKT/mTOR pathway was achieved via the soluble EpEX or cytoplasmic EpICD. It is possibly needed to specifically delineate in the future studies that EpCAM supports cancer cells growth either via the soluble EpEX or cytoplasmic EpICD as it is already known that these two domains are both pro-oncogenic. In addition, this knowledge could be valuable in the development of EpCAM therapeutic agents. EpCAM localization and proposed mechanism of actions in cancer are summarized in Figure 4.

## 6. Additional Insights from EpCAM Loss-of-Function Studies

There were several studies that have utilized the transgenic EpCAM knockout mouse models to further investigate the functional roles of EpCAM in health and diseases. Nagao et al. [113] generated EpCAM -/- knockout mice that resulted in embryonic lethality at E12.5 and suffered from developmental abnormalities. Other studies have shown that a small proportion of EpCAM homozygous knockout mice exhibited intestinal defects and impaired structural integrity of tight junctions, which eventually leads to death [52,114,115]. EpCAM mutants with deletion of its exon 4 (Δ4/Δ4) mice showed decreased expression of tight junctional proteins that leads to ion transport dysfunctionality in the intestines [116]. All the above studies utilized gene targeting to generate AGR2 knockout mice. Yang et al. recently generated AGR2 knockout mice using the CRISPR/Cas9 technology by targeting exon 2 of EpCAM [117]. The results resemble those mice generated using gene targeting in which the majority of the mice (42/47) demonstrated neonatal lethality after 1 week of birth, demonstrated intestinal anomalies and reduced the expression of hepatic glycogen-related genes. Altogether, this suggests that EpCAM expression is essential especially during embryogenesis and neonatal development. This growth stimulating property of EpCAM in mice early development sets a paradigm and foreshadows its role in human cancer cell proliferation, invasion and metastasis.

Another biological insight of EpCAM functions came from in-vivo studies using zebrafish and Xenopus model. EpCAM knockout zebrafish displayed defects in epithelial morphogenesis and integrity during epiboly and skin development [118]. This is in agreement with most in-vitro studies that suggests EpCAM is important in maintaining epithelial barrier and is further indicative of how it can function in disease like cancer to promote pro-metastatic properties. Two studies using EpCAM morpholino-mediated knockdown in Xenopus models further supported the involvement of EpCAM in regulating adherens junctions and E-cadherin integrity [56,119]. A different study using zebrafish EpCAM null mutant demonstrated that the mutant exhibited liver impairment via modulation of endoderm-specific WNT signalling through its EpEX domain (i.e., directly binds to Kremen1) [120].

There were also studies in mammalian cell lines utilizing genome editing tools to study EpCAM loss-of-function. For instance, one study generated single-cell clones of CRISPR/Cas9 mediated EpCAM knockout using E14TG2α embryonic stem cells markers [61]. The clones showed reduced expression of pluripotency genes such as Oct3/4, Sox2 and Nanog and displayed significant >50% reductions of endodermal markers. As described previously, CRISPR-Cas9 mediated EpCAM knockout in FaDu hypopharynx carcinoma cell line showed no notable effect on cell-matrix or cell-cell adhesion [58]. However, another study that generated EpCAM and its paralog TACTSTD2 double knockout in human corneal epithelial HCE-T cells using Transcription Activator-Like Effector Nuclease (TALEN) technology exhibited a significant reduction in epithelial barrier function [121]. Additionally, the double knockout cells altered sub-cellular localization of CLDN1 and CLDN7 proteins in which the proteins were translocated from plasma membrane to cytoplasm. Hence, this suggests relatively indirect role of EpCAM in regulating EpCAM in cell-cell adhesion. It may be concluded that EpCAM has biphasic effects, either promoting or attenuating cell-cell adhesion.

## 7. Discussion and Concluding Remarks

EpCAM has been widely associated with the ‘epithelial’ marker since its expression is abundant in both normal and malignant epithelial cells [5,14,49]. Its overexpression, high immunogenicity in cancers, coupled with pro-oncogenic features have enabled EpCAM as a pathophysiologically relevant anti-tumour target [4,6,7]. Several monoclonal antibodies have been developed, but these are largely targeting the large extracellular stalk [7]. Some of the antibodies targeting EpCAM has not shown consistent benefits in clinical trials [122]. EpCAM-specific monoclonal antibody edrecolomab for instance, while it showed good efficacy in early clinical trials, its benefits could not be replicated in larger clinical trials in treating patients with colon cancer [123,124]. A different mechanism of action needs to be revised to maximise the targeting EpCAM. For example, since EpCAM has oncogenic intracellular signalling function, would it be possible and effective to target intracellular domain of EpCAM in order to eradicate cancer? Targeting the intracellular protein is now possible with the advent of drug delivery technologies [125,126]. It is also important to note that targeting EpCAM may need fine-tuning as to find the best window since EpCAM is also expressed in normal epithelia and is important in embryogenesis, and more importantly this is to prevent adverse systemic effects [122]. The core function of EpCAM as a mediator of homophilic cell adhesion has been challenged recently, as some researchers could not find a direct involvement of EPCAM in mediating cell-cell contacts [31,58]. Additionally, the ‘CAM’ suffix confuses with the current CAM family that responsible for the majority of cell adhesion activities, hence EpCAM function is clouded with this CAM definition [2,4]. To avoid misconception, some researchers suggested that the molecule name should be revised to Epithelial Cell “Activating” Molecule [4,31,58]. Nevertheless, EpCAM remains a significant mediator of cell-cell contacts since in-vitro and in-vivo studies showed that it is involved in maintaining epithelial integrity via interaction with claudin proteins [51,53,121].

EpCAM can be found in the bodily fluid as it was expressed in CTC and exosomes isolated from the blood of cancer patients [70,101,104]. A high count of CTCs expressing EpCAM has been associated with poor outcome in cancer patients with or metastasis [127,128,129,130]. Additionally, there are some CTCs that have low or are negative for EpCAM, and therefore the inclusion of another CTC marker as a target may be needed to maximise therapeutic efficiency [80,131]. Only a certain fraction of CTCs can survive the blood circulation and travel to a distant site and these persisting cells are called disseminated tumour cells (DTCs) [132]. Some DTCs may stay dormant but some proceed toward metastases. It is thought that the microenvironment plays a role in determining the fate of these cells and regulate their growth. EpCAM is one of the established markers of tumour initiating cells that have the capability to renew itself, enhance replicative properties and support pluripotency [133]. Understanding the role of EpCAM in the tumour niche is another aspect of research that can be explored that may add supplementary knowledge to the biology of EpCAM.
